# State Clustering of the Hot Strip Rolling Process via Kernel Entropy Component Analysis and Weighted Cosine Distance

**DOI:** 10.3390/e21101019

**Published:** 2019-10-21

**Authors:** Chaojun Wang, Fei He

**Affiliations:** Collaborative Innovation Center of Steel Technology, University of Science and Technology Beijing, Beijing 100083, China; G20179048@xs.ustb.edu.cn

**Keywords:** hot strip rolling, kernel entropy component analysis, weighted cosine distance, recursive-based regional center

## Abstract

In the hot strip rolling process, many process parameters are related to the quality of the final products. Sometimes, the process parameters corresponding to different steel grades are close to, or even overlap, each other. In reality, locating overlap regions and detecting products with abnormal quality are crucial, yet challenging. To address this challenge, in this work, a novel method named kernel entropy component analysis (KECA)-weighted cosine distance is introduced for fault detection and overlap region locating. First, KECA is used to cluster the training samples of multiple steel grades, and the samples with incorrect classes are seen as the boundary of the sample distribution. Next, the concepts of recursive-based regional center and weighted cosine distance are introduced. For each steel grade, the regional center and the weight coefficients are determined. Finally, the weighted cosine distance between the testing sample and the regional center is chosen as the index to judge abnormal batches. The samples in the overlap region of multiple steel grades need to be focused on in the real production process, which is conducive to quality grade and combined production. The weighted cosine distances between the testing sample and different regional centers are used to locate the overlap region. A dataset from a hot steel rolling process is used to evaluate the performance of the proposed methods.

## 1. Introduction

With the constant increase of market demands for high-quality products, process monitoring and state evaluation technology become more and more attractive [1,2,3]. It is of great economic value to monitor the production process in real-time. In order to meet the different requirements in various applications, the demand for customized production has been augmented and the product types have been refined. The industrial production process gradually translates from mass production to high-mix, low-volume production.

Mechanical properties are important indicators of the final quality of hot rolled strips [4], which are affected by the chemical composition, temperature, and thickness of the hot strip, and many other important process parameters [5,6]. However, it is difficult to establish an accurate mechanical properties model since it is a highly automated and complex production process. To reduce the cost, people usually test a small number of products via sampling. Meanwhile, the inspection experiment is taken every several hours so that the result of product quality can be obtained in a timely manner. Thus, Wang established a novel neural network (NN) called PSO-BP NN, which combined the particle swarm optimization (PSO) and the back propagation (BP) rapid training algorithm to predict the mechanical properties [7]. Zhao introduces a semi-parametric single-index model to predict the mechanical properties of hot rolled strip [8]. It is a practical industrial problem that has to be solved urgently. Furthermore, with continuous maturity of production technology, the rate of unqualified products drops sharply. From a large amount of historical production data, it is concluded that there is sometimes an overlap in the process parameters of different steel grades, which is of great significance for quality control. In a real production process, the phenomenon of steel grade change and merger production is frequent, and it is particularly important to make rational use of this overlap. Therefore, how to judge the quality of products correctly and control the final quality according to the quality-related process parameters become important research topics.

In this paper, a clustering algorithm is employed to classify the datasets from different steel types, in which the data with incorrect classes are seen as the hypersurface since their process parameters deviate from the theoretical values. Kernel entropy component analysis (KECA) is first used to extract nonlinear characteristics via a kernel function [9,10,11]. Next, a distinct angular statistics method, named the Cauchy–Schwarz(CS) statistic, based on KECA is used to calculate the cosine value between batches [12]. In order to evaluate the mechanical properties of hot rolled steel offline more efficiently, KECA is used to extract features, and the kernel parameter and cluster number were selected adaptively to conduct the production state clustering [13]. KECA extracts features via Renyi entropy, and can achieve a better feature extraction performance than kernel principal component analysis (KPCA) [14]. However, the above work only focuses on the fault detection of one steel grade while the overlap region is not taken into consideration. Another entropy-based clustering approach is cross-entropy clustering [15]. The improvement of multi-objective optimization based on cross-entropy method is introduced in [16,17]. To enhance the cluster analysis, an information-theoretic-based method for cluster visualization using self-organizing maps (SOMs) is presented [18]. 

Support vector data description (SVDD) is derived from the support vector machine (SVM) and has been widely used in process monitoring. It focuses on determining a hypersphere with minimum volume to describe the characteristics of target classes [19]. In recent years, some researchers have applied SVDD in batch process monitoring [20,21,22]. Meanwhile, it is also widely applied in data classification [23,24,25] and outlier detection [26,27]. The SVDD method builds a hypersphere based on the training dataset to cover as many datasets as possible and minimize its volume. Similarly, a hypersurface is built in a high-dimensional space to distinguish normal and abnormal batches, where the batches inside the hypersurface are seen as normal. In this work, we consider not only the abnormal samples but also the overlap region of multiple steel grades. This paper introduces the recursive-based regional center, which is defined as a region that the weighted cosine distance from it to the boundary of the data distribution along different directions is a constant. Obviously, the constant is the radius of the hypersurface. The cosine distance is the index to distinguish normal and abnormal batches. First, the information of the data distribution is determined via the KECA algorithm. The training datasets are divided into two parts according to the data distribution, where the first part is on the boundary and the other part is inside the boundary. Then, the recursive-based regional center is determined. Finally, the test datasets are detected via the cosine distance. In order to control the quality of steel better, it is worth paying attention to the overlap of the process parameters of multiple steel grades. The weighted cosine distances between the test dataset and different regional centers are also used to judge which dataset is in the overlap.

The rest of this paper is organized as follows: Section 2 introduces the KECA clustering algorithm. In Section 3, we present a novel method named the KECA-weighted cosine distance for abnormal sample detection and overlap region locating. Section 4 validates the performance of our proposed method using numerical studies. Finally, Section 5 concludes the paper.

## 2. Kernel Entropy Component Analysis 

### 2.1. Kernel Entropy Component Analysis

Consider a dataset $X:x_{1},x_{2}\cdots x_{n}$ with the probability density function p(x). The Renyi quadratic entropy is defined as [28]:

(1) $$\overset{\wedge }{V}(p)=-\log(V(p))=-\log(\int p^{2}(x)dx)$$

and the Parzen window density estimator is described as:

(2) $$\overset{\wedge }{p}(x)=\frac{1}{N}\sum_{x_{i}\in X}k_{\sigma }(x,x_{i})$$

where $k_{\sigma }(x,x_{i})$ is a Mercer kernel function, and *σ* is the parameter of the kernel function, or Parzen window. Using the sample mean approximation of the expectation operator, we can express the Renyi quadratic entropy as follows:

(3) $$\overset{\wedge }{V}(p)=\frac{1}{N}\sum_{x_{i}\in X}\frac{1}{N}\sum_{x_{i}\in X}\int k_{\sigma }(x,x_{i})k_{\sigma }(x,x_{i})dx,$$

which can be re-expressed as follows:

(4) $$\overset{\wedge }{V}(p)=\frac{1}{N^{2}}{Ι}^{Τ}ΚΙ$$

where **K** is the kernel matrix and **I** is an (*n* × 1) vector where each element equals one. The kernel matrix can be decomposed as $Κ=\Phi^{Τ}\Phi =\mathrm{EDE}$ using eigen decomposition, where D is a diagonal matrix storing the eigenvalues $\lambda_{1},\lambda_{2},\cdots ,\lambda_{N}$ and **E** is a matrix with the eigenvectors $e_{1},e_{2},\cdots ,e_{N}$ as columns. Therefore, the Renyi entropy estimator in Equation (3) can be expressed using the eigenvalues and eigenvectors:

(5) $$\overset{\wedge }{V}(p)=\frac{1}{N^{2}}\sum_{i=1}^{N}{\left(\sqrt{\lambda_{i}}e_{i}^{T}I\right)}^{2}$$

Equation (5) demonstrates that each eigenvalue coupled with its corresponding eigenvector make a different contribution to the Renyi quadratic entropy. In this paper, the *t* eigenvalues that make the first *t* largest contributions to the Renyi quadratic entropy will be selected, which make the contributions greater than 85%. This yields a component matrix $\Phi_{eca}=D_{t}^{\frac{1}{2}}E_{t}^{T}$.

### 2.2. Cauchy–Schwarz (CS) Statistic

There is a distinct angular structure in the feature space of KECA, in which different clusters are usually located in different angular directions. Thus, it is necessary to calculate proper statistics on the feature space. To express the angular structure of a dataset, the CS divergence measurement between the probability density functions, which corresponds to the cosine of the angle between kernel feature space mean vectors, has been derived. Then, the clustering steps based on KECA are given as follows:

(1) Obtain $\varphi_{eca}$ by KECA, and rank the eigenvectors $e_{1},e_{2},\cdots ,e_{N}$;
(2) Initialize means $m_{k},k=1,\cdot \cdot \cdot ,C$, C is the cluster number;
(3) For all $t:x_{t}\to C_{k}:\max\cos∠(\varphi_{eca}(x_{t}),m_{k})$;
(4) Update mean vectors;
(5) Repeat steps (3) and (4) until convergence.

In summary, we assign a data point $\varphi_{eca}(x_{t})$ to the cluster represented by the closest mean vector $m_{k}$ in terms of angular distance. In step 2, we initialize the mean vectors such that the two data points $\varphi_{eca}(x_{t})$ and $\varphi_{eca}(x_{t^{\prime }})$, having the property that $\min_{t,t^{\prime }}\cos∠(\varphi_{eca}(x_{t}),\varphi_{eca}(x_{t^{\prime }}))$, are selected to represent $m_{1}$ and $m_{2}$. Furthermore, $m_{i},i>2$ is represented by $\varphi_{eca}(x_{t^{\prime \prime }})$, such that $\min_{t^{\prime \prime }}\sum_{j=1}^{i-1}\cos∠(\varphi_{eca}(x_{t^{\prime \prime }}),m_{j})$. Here, $m_{i}=\frac{1}{N}\sum_{x_{n}\in D_{i}}\varphi_{eca}(x_{n})$ is the kernel feature space cluster mean vector corresponding to $C_{i}$, and $m=\frac{1}{N}\sum_{x_{t}\in D_{i}}\varphi_{eca}(x_{t})$ is the overall kernel space mean vector of the data. Based on our experience, this algorithm is robust to the initialization and usually converges within a few iterations. Convergence is measured by the change in a CS cost function from one iteration to the next [29].

The CS divergence measures the cosine of the angle between these mean vectors. An angle-based clustering cost function in terms of the kernel feature space dataset Φ may be defined as:

(6) $$J(C_{1},\cdot \cdot \cdot ,C_{c})=\sum_{i=1}^{c}N_{i}\cos∠(m_{i},m)$$

## 3. Process Parameters Clustering via Weighted Cosine Distance

From the quality control perspective, we are interested in identifying the abnormal batches and overlap regions. To achieve this goal, we employ KECA for multiple steel grades clustering. KECA provides the information of the data distribution, which can be used for locating the overlap region. The weighted cosine distance is a novel distance measurement in the high-dimensional space and can be used as a metric for abnormal sample monitoring and locating the overlap region.

### 3.1. Weighted Cosine Distance

In this section, a weighted cosine distance is proposed to measure the distance in the high dimensional space between a sample and other samples. 

Consider the datasets $X_{r}(N\times M)$ with N samples and M variables. The CS statistic matrix S(N×N) is a symmetric matrix, in which each element $s_{mn}$ is the cosine value between the *m-th* sample and the *n-th* sample. Obviously, the cosine values range from –1 to 1. The closer the cosine values is to 1, the shorter the distance. In order to compare the distance conveniently, the cosine distance is defined as follow:

(7) $$s_{mn}=\frac{x_{m}\cdot x_{n}}{\left\|x_{m}\right\|\left\|y_{n}\right\|}$$

(8) $$d_{mn}={\left(1-s_{mn}\right)}^{2}(m=1,\ldots ,N,n=1,\ldots ,N)$$

where, $d_{mn}$ is the element of the cosine distance matrix D and it represents the distance between the *m-th* sample and the *n-th* sample. The range of cosine distance is enlarged via the square operation. Obviously, the cosine distance ranges from 0 to 4.

Sometimes, many samples need to be considered as a whole. The new distance model named the One–Many cosine distance is introduced. Consider one sample x(1×M) and a dataset X(N×M), where the dataset X contains N samples. For each samples in the dataset X, we calculate its cosine value with sample x. As a result, a vector s(1×N) is obtained. The One-Many cosine distance is defined as

(9) $$d=\frac{1}{N}\sum_{i=1}^{N}{\left(1-s_{i}\right)}^{2}$$

In fact, the role of the samples in the datasets X(N×M) may be different. Thus, when calculating the One–Many cosine distance, the weights of different samples should be considered instead of the average. Assume a weight coefficient vector $\alpha (\alpha_{1},\alpha_{2},\cdots ,\alpha_{N})$ is given, where $\alpha_{i}$ is the weight coefficient of the *i*-*th* sample, the weighted cosine distance is defined as follows:

(10) $$d=\frac{1}{N}\sum_{i=1}^{N}\alpha_{i}{\left(1-s_{i}\right)}^{2}$$

### 3.2. Abnormal Samples Detection

**Figure 1:**
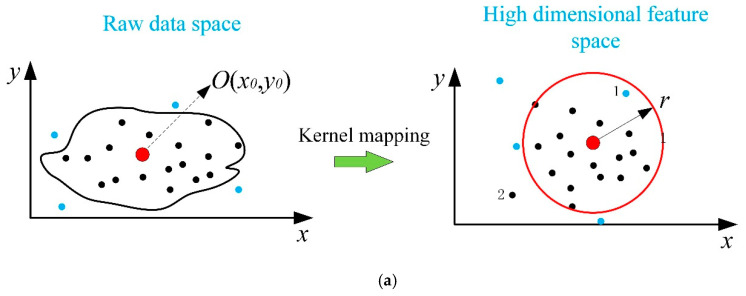

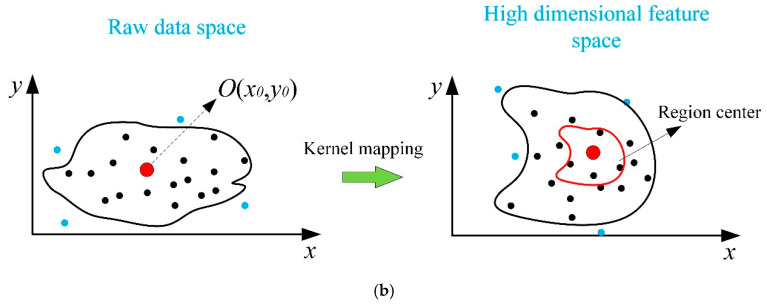
Comparison of two methods. (**a**) Schematic diagram of the traditional fault detection method; (**b**) schematic diagram of the recursive based regional center.

The samples are projected into high-dimensional feature space via the kernel function. The hypersphere is built in the high dimensional feature space. If the sample is inside the hypersphere, it is seen as normal, otherwise it is seen as abnormal. As shown in Figure 1a, the blue points represent the abnormal samples and the black points represent the normal samples. Assume *O*(*x*_0_,*y*_0_) is the center of the hypersphere. If the hypersphere is selected with the radius r, some samples are misclassified. For example, sample 1, which is abnormal, is inside the hypersphere, that is, it is misjudged as normal. Another example is sample 2, which is misclassified as abnormal.

Assume that there is such a hypersurface in the high-dimensional feature space that contains all the normal samples, and all the abnormal samples are outside the hypersurface. No doubt, this hypersurface can replace the hypersphere and classify the process state more accurately. In order to overcome the problems caused by the hypersphere, this paper introduces a recursive-based regional center method. It defines as a region in high-dimensional feature space, which has the same distance from it to the hypersurface along different directions. As shown in Figure 1b, the regional center enlarged from the sample *O* to the regional center that is considered as a whole. The new radius *R* is defined as the mean of the cosine distances between the samples in the regional center and the hypersurface. Obviously, the choice of the regional center is the key problem. Thus, a recursive method is proposed.

However, it is difficult to determine a qualified region based on the irregular distribution in the high-dimensional feature space. Obviously, different samples in the regional center make different contributions to the distance calculation. Thus, the weight coefficients are selected to quantify the contribution of each sample in distance calculation. It is worth noting that both the radius and the weight coefficients are unknown. If the first one of them is set as a constant value, then we can obtain the other one via a recursive method and update the first one. The computation will loop continuously until convergence. To ensure that the regional center meets its definition, the radius r must be a constant that is given firstly, the weight coefficients can be obtained via regression. Thus, the regression process can calculate as follows:

(11) $$r=\frac{1}{N}\sum_{i=1}^{N}\alpha_{i}{\left(1-s_{i}\right)}^{2}+\varepsilon$$

where $\alpha (\alpha_{1},\alpha_{i},\cdots ,\alpha_{N})$ is the weight coefficients, ε is the residual. $s_{i}$ is calculated by Equation (7) where s is a vector when the regional center is selected, and all the samples in the center are seen as a whole.

Consider two datasets $X(x_{1},x_{2},\cdots ,x_{N_{1}})$ and $Y(y_{1},y_{2},\cdots ,y_{N_{2}})$ with $N_{1}$ and $N_{2}$ samples, respectively, and M variables, where dataset X is the regional center and dataset Y is distributed on the hypersurface. According to Equation (11), it can be further obtained that:

(12) $$r_{n}=\frac{1}{N_{1}}\sum_{m=1}^{N_{1}}\alpha_{m}{\left(1-s_{mn}\right)}^{2}+\varepsilon_{n}(n=1,2,\cdots ,N_{2})$$

where $\alpha (\alpha_{1},\alpha_{2},\cdots ,\alpha_{N_{1}})$ are the weight coefficients; $r(r_{1},r_{2},\cdots ,r_{N_{2}})$ is the radius vector whose elements are set as one value r. This implies that the distance between the samples on the hypersurface and the regional center is a constant; $s_{mn}$ is the cosine value between the *m*-*th* sample in dataset X and the *n*-*th* in dataset Y; $\varepsilon (\varepsilon_{1},\varepsilon_{2},\cdots ,\varepsilon_{N_{2}})$ is the residual vector. To guarantee the given radius stable, its residual ε must be stable. Thus, it is necessary to minimize the variance of residuals.

To achieve abnormal samples detection, the recursive-based regional center should be determined firstly. Consider two datasets $X_{1}(x_{1}^{1},x_{2}^{1},\cdots ,x_{I_{1}}^{1})$ and $X_{2}(x_{1}^{2},x_{2}^{2},\cdots ,x_{I_{1}}^{2})$ with $I_{1}$ and $I_{2}$ samples, respectively, and *J* variables, where dataset $X_{1}$ is inside the hypersurface and dataset $X_{2}$ is the boundary of the hypersurface. Calculate the cosine value matrix according to Equation (7) and take the sum of them as follows:

(13) $$T=\sum_{n=1}^{I_{2}}s_{mn}(m=1,2,\cdots ,I_{1})$$

Then, sort the vectors T in ascending order. Obviously, the smaller the sum of the cosine values, the closer it is to the regional center. The detailed procedure of the recursive-based regional center is described as Algorithm 1 below. 


**Algorithm 1: Recursive-based regional center**
 Step 1. Calculate the sum of the cosine values T according to Equation (13); Step 2. Reconstruct the datasets $X_{1}$ according to the ascending order of the vector T; Step 3. Let k=1, the given radius is selected as r; Step 4. Select regional center $C=[x_{k}^{1}]$, Calculate the residual vector ε according to Equation (12); Step 5. Let ovar = var(ε); Step 6. Do while $k\leq I_{1}$      *k*=*k*+1; C=[C;$x_{k}^{1}$];      nvar = var($\varepsilon^{\prime }$), where $\varepsilon^{\prime }$ is the residual in Equation (12);      if ovar ≥ nvar       ovar = nvar;       continue;      else       remove $x_{k}^{1}$ from C;      end

Last, the matrix C **is** the regional center. The weighted coefficients α is obtained according to Equation (12). Thus, the weighted cosine distance between each testing batch and the regional center is calculated. If the distance is larger than the given radius r, it means the data sample is outside the hypersurface and is seen as abnormal. Otherwise, it is normal.

### 3.3. Locating the Overlap Region 

As is known, many steel grades have similar mechanical properties due to their close process parameters. Sometimes their process parameters appear in an overlap region. Obviously, it is of great significance to make full use of the overlap region.

**Figure 2:**
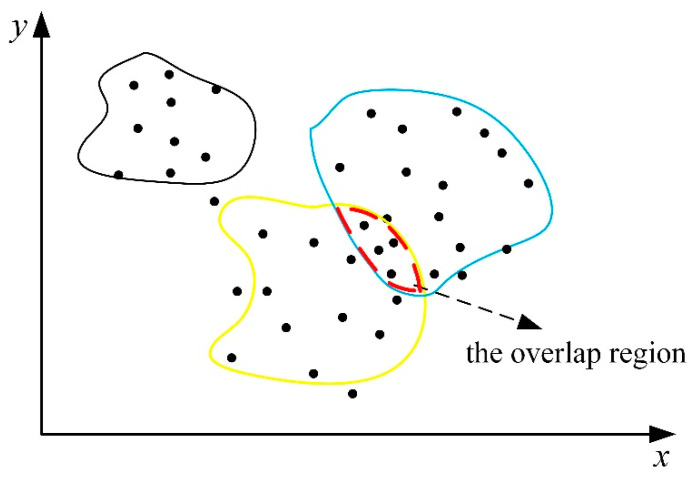
Schematic diagram of data distribution for different steel types.

The schematic diagram of the data distribution from different steel types is shown in Figure 2, where black points are the datasets, and the lines of the three colors (black, blue, and yellow) represent the hypersurface composed of three steel grades in the high-dimensional space. If the mechanical properties of two steel grades are similar, the process parameters may still overlap in the high-dimensional space, for example, the area formed by the red dotted line. Assume three colors (black, blue, and yellow) represent three steel grades, steel 1, steel 2, and steel 3, respectively. Obviously, steel 1 is far away from the other steel grades, so there will be no overlap region. However, steel 2 is similar to steel 3. Despite the datasets in the overlap region being normal, the process parameters have deviated from their theoretical values. In fact, the datasets in the overlap region are also worth discussing since it can be used for steel grade change and production grade merger. 

For each steel grade, the regional center is determined based on the recursive method. The weighted cosine distance between the datasets in the overlap region and the regional center of steels 2 and 3 is less than the given radius r (the given radius can be the same for different steel grades). This characteristic is the necessary and sufficient condition for judging whether a sample is inside the overlap region.

### 3.4. Procedure of Process State Clustering

**Figure 3:**
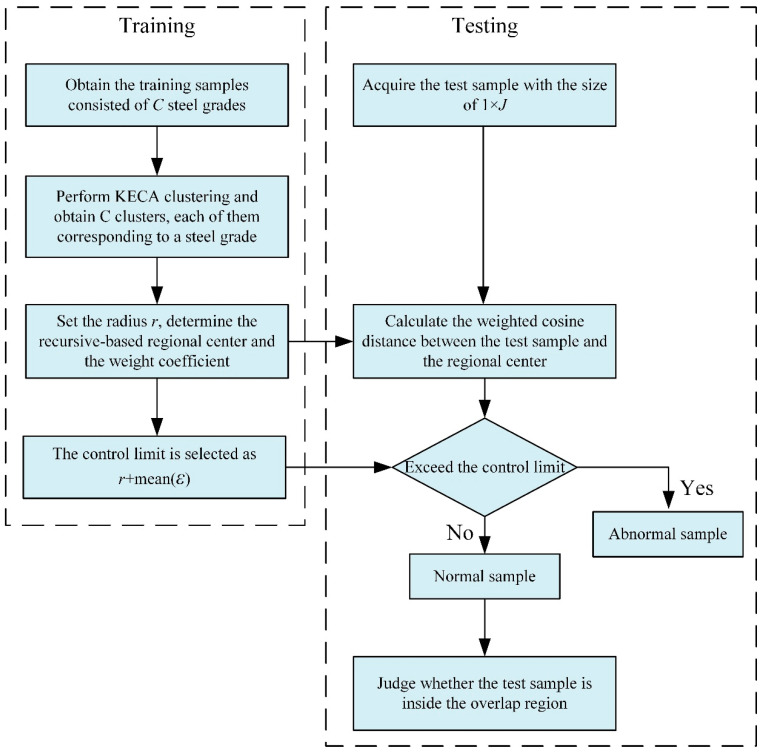
Flowchart of the proposed method.

The procedure of the proposed method is shown in Figure 3. In the training process, the training datasets with the size of *I*×*J* are obtained, which contain *C* steel types. The KECA clustering is used to divide the training dataset into *C* clusters. The cluster number C is the number of steel grades. If the original number of steel grades is unknown, the optimal number of clusters can be determined by a novel cluster validity index (CVI) that is introduced for cluster validation [30]. Then, the dataset of each steel grade is divided into two parts according to the clustering result. The first part with correct clusters is seen as inside the hypersurface, and the second part with incorrect clusters is considered as the hypersurface. Then, we set the given radius as r. The recursive-based region center and the weight coefficients are determined. The procedure of process state clustering via the KECA-weighted cosine distance is summarized below.

Off-line training process:

(1) Obtain the training samples $X(X_{1},\cdot \cdot \cdot ,X_{C})$ from historical normal operating conditions, where *C* represents the number of steel types;
(2) Perform KECA clustering on datasets **X**, and then *C* clusters are obtained. For each product $X_{k}(I\times J)$, it is divided into two parts, $X_{k}^{r}(I_{1}\times J)$ and $X_{k}^{inr}(I_{2}\times J)$ that are seen as the hypersurface;
(3) Set the given radius as r; the recursive-based regional center and the corresponding weight coefficients are determined;
(4) Set the control limit as r+mean(ε).

On-line fault detection:

(1) Acquire the testing sample with the size of 1 × *J*;
(2) Calculate the weighted cosine distance between the recursive-based regional center and the testing sample;
(3) The weighted distance is compared with the control limit; if it exceeds the control limit, it would be judged as abnormal. Otherwise, it would be considered as normal;
(4) For the normal sample, calculate the weighted cosine distances between it and the regional center of other steel types. If one or more weighted cosine distances are less than the control limit, it is inside the overlap region.

## 4. Experiments

In this section, a dataset from a hot strip rolling process is employed to demonstrate the effectiveness of the KECA-weighted cosine distance method. The proposed method is compared with the method ClusterSVDD [31] and the detailed results are described. 

**Figure 4:**
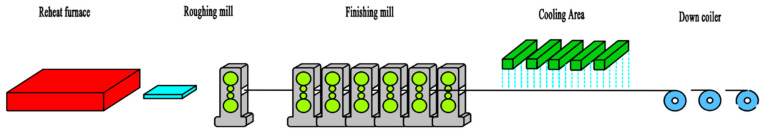
Layout drawing of hot rolling process.

The hot strip rolling production is a complex industrial process with the purpose of turning reheated steel slabs into strips. The layout drawing of the hot rolling process is shown in Figure 4 [32]. The commonly hot strip rolling line consists of several major pieces of equipment, such as reheat furnaces, a roughing mill, a transfer table, a finishing mill, laminar cooling, and coilers. In the rough rolling process, the thickness of the reheat steel slabs is reduced quickly. To satisfy the requirements of the reduction, final qualities, and tolerances, steel plants execute the rolling process of tandem rolling with six or seven successive stands during the finishing rolling process in which the reheated steel slabs are narrowed into the final thickness via the repeated rolling process.

Mechanical performance is a significant quality of the final product. There are 11 process variables that are most related to the mechanical performance, and which are collected during the real process, as shown in Table 1. 

The real production data of three steel grades are used to illustrate the proposed method. A total of 65 samples per steel grades are collected as the training dataset. Thus, there are 195 samples with a size of 195 × 11. For the first steel grade, a total of 79 samples are selected as the testing dataset, which contains 14 fault samples and 65 normal samples. There are 87 samples consisting of 22 fault samples and 65 normal samples, seen as the testing datasets of the second steel grade. The third steel grade contains 81 samples where 16 are fault samples and 65 are normal samples.

Due to the imbalance of classes in the testing dataset, that is, there is a large difference in the proportion of normal and abnormal samples, the detection rate, ROC criteria, and AUC value are employed to evaluate different models. The detection rate is defined in Equation (14). The horizontal axis of the ROC is the false positive rate (FPR) and the vertical axis is the true positive rate (TPR). The area under the curve (AUC) is defined as the area under the ROC curve:

(14) $$rate=\frac{TP+TN}{TP+TN+FP+FN}$$

(15) $$FPR=\frac{FP}{FP+TN}$$

(16) $$TPR=\frac{TP}{TP+FN}$$

where *TP* represents the number of normal samples which are judged as normal; *FP* represents the abnormal samples which are judged as normal; similar, *FP* is the number of normal samples that are detected as abnormal; and *TN* is the number of abnormal samples that detected as abnormal.

The Gaussian kernel $k(x,x^{\prime })=\exp(-{\left\|x-x^{\prime }\right\|}^{2}/c)$ that is widely used in the field of process monitoring is employed to project the datasets into the high-dimensional feature space. Different parameters are selected to build the best models for each method, such as the given radius r, the kernel parameter *c* ∈ {10^–3^, 10^–2^, …, 10^7^}, and so on.

The clustering results via KECA are shown in Table 2. As we know, KPCA and KECA are very similar [12,33] dimension reduction methods. In order to verify the effectiveness of KECA, the clustering results via KPCA are shown in Table 3. Both the best kernel parameters of KECA and KPCA are selected as 10^2^, which range from 10^3^ to 10^7^. Obviously, the results of KECA are better than the results of KPCA. In fact, how to select the features is the most important difference between KPCA and KECA. KPCA is subject to the assumption of a normal distribution of data, which selects the principal components according to the variances. However, industrial data has unpredictable complexity that is very difficult to obey the normal distribution. KECA has a clear theoretical foundation based on the concept of maximum entropy preservation. The novel strategy based on information entropy avoids the distribution assumption of data and chooses the information as the index directly. Therefore, it has better universality in industrial application. 

According to the clustering results via KECA, the third steel grade is recognized correctly while the first two steel grades have some samples with incorrect results. Obviously, the third steel grade is different from the first two steel grades. To confirm this, the mean of each process parameter for the three steel grades are shown in Table 4. The process parameters, such as the content of P, the entry temperature of the roughing milling and finish milling, the exit temperature of the finish milling, and the coiling temperature are different from other steel grades. Thus, the third steel grade overlap with the first two steel grades. 

**Figure 5:**
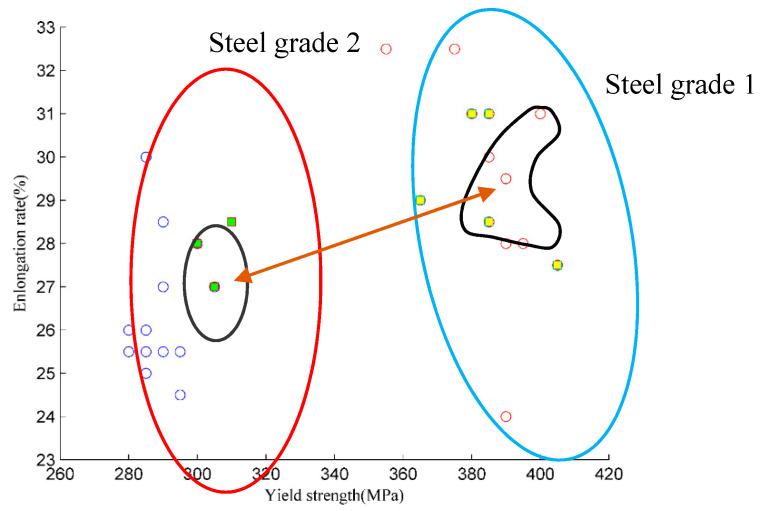
The yield strength–elongation rate diagram.

The scatter plot between yield strength–elongation of the first two steel grades is shown in Figure 5. The samples in the blue curve are from steel grade 1; red hollow circles represent the samples with correct clusters, and yellow solid circles are these samples with incorrect clusters. The samples in the red curve are from steel grade 2; blue hollow circles represent the samples with correct clusters and green solid rectangles are those samples with incorrect clusters. In Figure 5, there are 130 batches. A large number of samples are shown in the same position because they have the same values. Although the KECA is performed on the process parameters, the distribution of the multivariable process parameters cannot be shown via visualization methods. Therefore, the clustering results can only be shown by their mechanical properties. For these samples with incorrect clusters, even though their mechanical properties do not appear in the overlap region, their process parameters deviate from their theoretical value, which needs focus in order to improve the quality of steel further. Thus, it is of great significance to focus on the overlap region between different steel grades.

The kernel parameter is selected via a grid search and then verified using cross-validation. In the proposed method, the kernel parameter is selected as 102. The given radius is 160. The number of clusters is three, as there are three different steel grades. There are a total of 11 process parameters selected and the number of principal components is five. In ClusterSVDD, the number of principal components is still five. The number of cluster is three and the kernel parameter is selected as 50. The kernel parameter is selected from the range of 10^−7^–10^3^. Similarly, the given radius is selected by the same way, which is in the range from 1 to 200.

**Figure 6:**
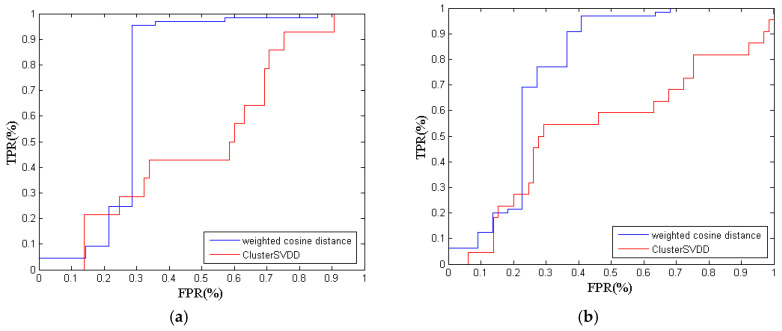

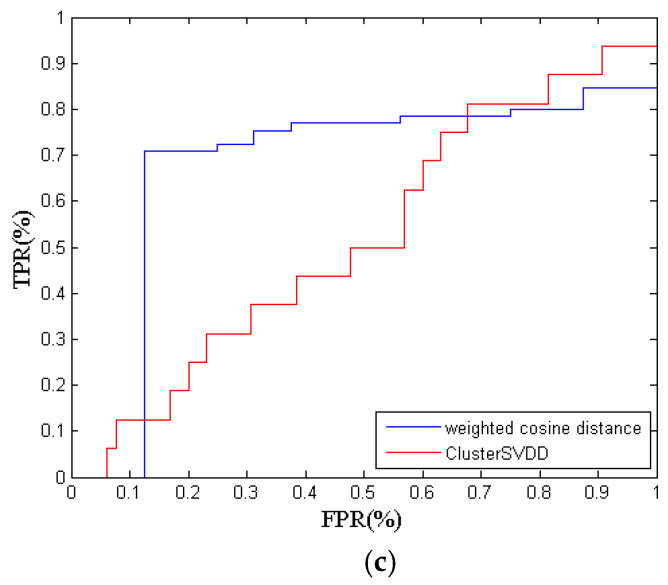
The ROC criteria of two models. (**a**) The first steel grade; (**b**) the third steel grade; (**c**) the third steel grade.

The detection results of the proposed method and the ClusterSVDD are presented in Table 5. For the first steel grade, the detection rate of the KECA-weighted cosine distance is 75.95%, while the detection rate of ClusterSVDD is only 51.90%. Obviously, the computational results show that the weighted cosine distance gives better monitoring performance. The ROC criteria are shown in Figure 6a. The AUC value based on the weighted cosine distance is 0.7308, while the ClusterSVDD returns the AUC value of 0.5287, which demonstrates that the proposed method is more accurate.

For the second steel grade, the weighted cosine distance returns the highest detection rate of 86.21%, while the result of ClusterSVDD is 51.72%. The ROC criteria are shown in Figure 6b. The KECA-weighted cosine distance gives better performance than ClusterSVDD. Meanwhile, the AUC value of the weighted cosine distance is 0.7559, which is larger than the AUC value of ClusterSVDD.

Similarly, the result of the proposed method for the third steel grade is still better than the result of ClusterSVDD. The ROC criteria are shown in Figure 6c. 

To verify the performance of the overlap region locating, a total of 33 samples are selected as the testing dataset. As mentioned above, the first two steel grades have an overlap region. All the weighted cosine distances from these samples to the two regional centers are less than the given radius r. The detection rate is 72.73%, that is, there are 24 samples that are detected correctly.

In summary, the results for three steel grades demonstrate that the KECA-weighted cosine distance gives better monitoring performance than ClusterSVDD. Furthermore, the KECA-weighted cosine distance works in this case where different products have similar process parameters.

## 5. Conclusions

This paper introduces two new concepts of the weighted cosine distance and recursive-based regional center. In addition, a novel method, the KECA-weighted cosine distance, is derived for the abnormal samples detection and overlap region locating. Unlike traditional methods, the proposed method enlarges the center into a region and calculates the weighted cosine distance. The data from a hot strip rolling process is used to evaluate the process monitoring performance. The detection results of the KECA-weighted cosine distance are compared with the results of ClusterSVDD, and the results illustrate that the proposed method gives better monitoring performance. Meanwhile, it can also locate the overlap region of different steel grades well. 

Some challenges are worth discussing in future works. For example, how to choose the given radius is worth considering and a meaningful topic. Furthermore, the dataset used to construct the hypersurface plays an important role, and an approach to determine the dataset is also an important topic to investigate in the future.

## Figures and Tables

**Table 1:** Variables table of process parameters.

	Type	Parameters Names	Number
Process parameters	Content of chemical components	C, Si, Mn, P, S	1–5
	Rolling process temperature	The entry temperature of roughing milling and finish milling, the exit temperature of finish milling and coiling temperature	6–9
	Rolling process thickness	The exit thick of roughing milling and finish milling	10–11

**Table 2:** The clustering results via KECA.

	Cluster 1	Cluster 2	Cluster 3
**Steel grade 1**	59	6	0
**Steel grade 2**	4	61	0
**Steel grade 3**	0	0	65

**Table 3:** The clustering results via KPCA.

	Cluster 1	Cluster 2	Cluster 3
**Steel grade 1**	49	11	4
**Steel grade 2**	12	52	3
**Steel grade 3**	2	4	58

**Table 4:** The mean of each process parameters for three steel grades.

	Steel Grade 1	Steel Grade 2	Steel Grade 3
C	0.1720	0.1631	0.1614
Si	0.1258	0.2018	0.1811
Mn	0.3934	1.2879	0.4985
P	0.0201	0.0194	0.0129
S	0.0112	0.0136	0.0129
The entry temperature of roughing milling	1059.3	1072.0	1100.6
The entry temperature of finish milling	997.3	976.7	1040
The exit temperature of finish milling	870	858.5	937.8
The exit temperature of coiling temperature	629	617.3	673.9
The exit thick of roughing milling	44.39	44.13	41
The exit thick of finish milling	8.06	5.72	7.96

**Table 5:** Fault detection rates and AUC values of the weighted distance and ClusterSVDD for the hot strip rolling process

Steel Grades	KECA-Weighted Cosine Distance		ClusterSVDD	
	Detection rate (%)	AUC	Detection rate (%)	AUC
**1.**	75.95%	0.7308	51.90%	0.5287
**2.**	86.21%	0.7559	51.72%	0.5276
**3.**	66.67%	0.6779	51.85%	0.5125
